# “FlashMap” - A Semi-Automatic Tool for Rapid and Accurate Spatial Analysis of Marker Expression in the Subventricular Zone

**DOI:** 10.1038/s41598-018-33939-1

**Published:** 2018-10-31

**Authors:** Stefan Zweifel, Julie Buquet, Lorenzo Caruso, David Rousseau, Olivier Raineteau

**Affiliations:** 1 Univ Lyon, Université Claude Bernard Lyon 1, Inserm, Stem Cell and Brain Research Institute U1208, 69500 Bron, France; 2 CREATIS CNRS UMR5220 & INSERM U1044, Université de Lyon, Université Claude Bernard-Lyon 1, INSA-Lyon, Villeurbanne, France; 3 Institut d’Optique, Université Paris-Saclay, 91127 Palaiseau cedex, France; 4 Télécom Physique Strasbourg, University of Strasbourg, Strasbourg, France; 5 LARIS, IRHS UMR INRA, Université d’Angers, 62 avenue Notre Dame du Lac, 49000 Angers, France

## Abstract

The subventricular zone (SVZ) is a region of ongoing postnatal germinal activity that shows complex spatial heterogeneity. For instance, different SVZ microdomains contain neural stem cells that express distinct transcription factors and generate different glial and neuronal progenies. These unique characteristics call for the development of new methods to integrate a spatial dimension to histological analyses performed in this germinal region. We developed “FlashMap”, a semi-automatic software that allows the segmentation and rapid measurement of optical densities throughout the full SVZ coordinates. “FlashMap” generates easily readable two-dimensional heatmaps that can be superimposed onto three-dimensional reconstructions of the ventricular system for optimal spatial exploration. Accurate heatmaps can be obtained, even following serial section subsampling thereby reducing the amount of tissue and time required for histological analysis. We first illustrate the potential of “FlashMap” by spatially exploring the correlation of SVZ thickness and cellular density with germinal activity throughout its rostro-caudal coordinates. We then used “FlashMap” to analyse the spatial expression of the transcription factors Dlx2, Tbr2 and Hopx as well as of the immature neuronal marker Dcx, to demonstrate the suitability of this approach to explore the regional production of cells of distinct lineages by defined SVZ microdomains.

## Introduction

The subventricular zone (SVZ) is a germinal region surrounding the opened lateral ventricles (LV). It is one of only two niches of the mammalian forebrain, where germinal activity persists throughout postnatal life^1–3^. At its apical border it lines the LV and is restricted at its basal borders by defined brain regions: i.e. the corpus callosum (dorsally), the striatum (laterally), the septum and hippocampus (medially). Neural stem cells (NSCs) harboured by the postnatal SVZ generate neuroblasts, that migrate along the rostral migratory stream (RMS) into the olfactory bulb (OB), where they differentiate into various types of neurons and integrate into pre-existing circuits^4,5^. Further, NSCs of the postnatal mammalian SVZ give rise to astrocytes and oligodendrocytes^6^. Accumulating evidences highlight the heterogeneous nature of the SVZ. Thus, the SVZ appears to be populated by regionalized populations of NSCs that are biased to generate distinct neuronal and glial subtypes^7^. For instance, most GABAergic (inhibitory) interneurons originate from the lateral microdomain of the SVZ (lSVZ), while progenitors of glutamatergic (excitatory) neurons are exclusively observed in its dorsal counterpart^8,9^. Similarly, oligodendrocytes are produced postnatally by the most dorsal regions of the SVZ^10^ (dSVZ). Similarly to rodents, dorsal restriction of glutamatergic progenitors and lateral enrichment of GABAergic progenitors was found in newborn marmosets^11^. This suggests that a certain degree of heterogeneity is evolutionary conserved.

Recent research, driven by the development of new technologies, led to an accumulation of a large amount of transcriptional datasets of various SVZ cell types isolated from distinct microdomains. For instance, we recently described an unexpected level of transcriptional heterogeneity between the dSVZ and lSVZ, as well as between NSCs and transient-amplifying progenitors of those two microdomains^12^. While the list of regionally expressed genes continuously increases, an appropriate tool for rapid analysis of their expression pattern in the SVZ is still missing. We therefore aimed to develop a tool that allows rapid protein expression analysis along the full rostro-caudal and dorso-ventral extend of this germinal region. “FlashMap” is a software for semi-automatic marker expression analysis in the SVZ, based on optical density (OD) measurements. “FlashMap” allows subsampling of serial sections, in order to further reduce the time for analysis. Data are exported as heatmaps that can be superimposed onto volumetric reconstructions of the LV for optimal and intuitive visualization. “FlashMap” has the potential to be applied to a wide range of semi-automated analyses, and is therefore of substantial interest for the field.

## Results

### The subventricular zone is a poorly defined region of the postnatal forebrain

**Figure 1:**
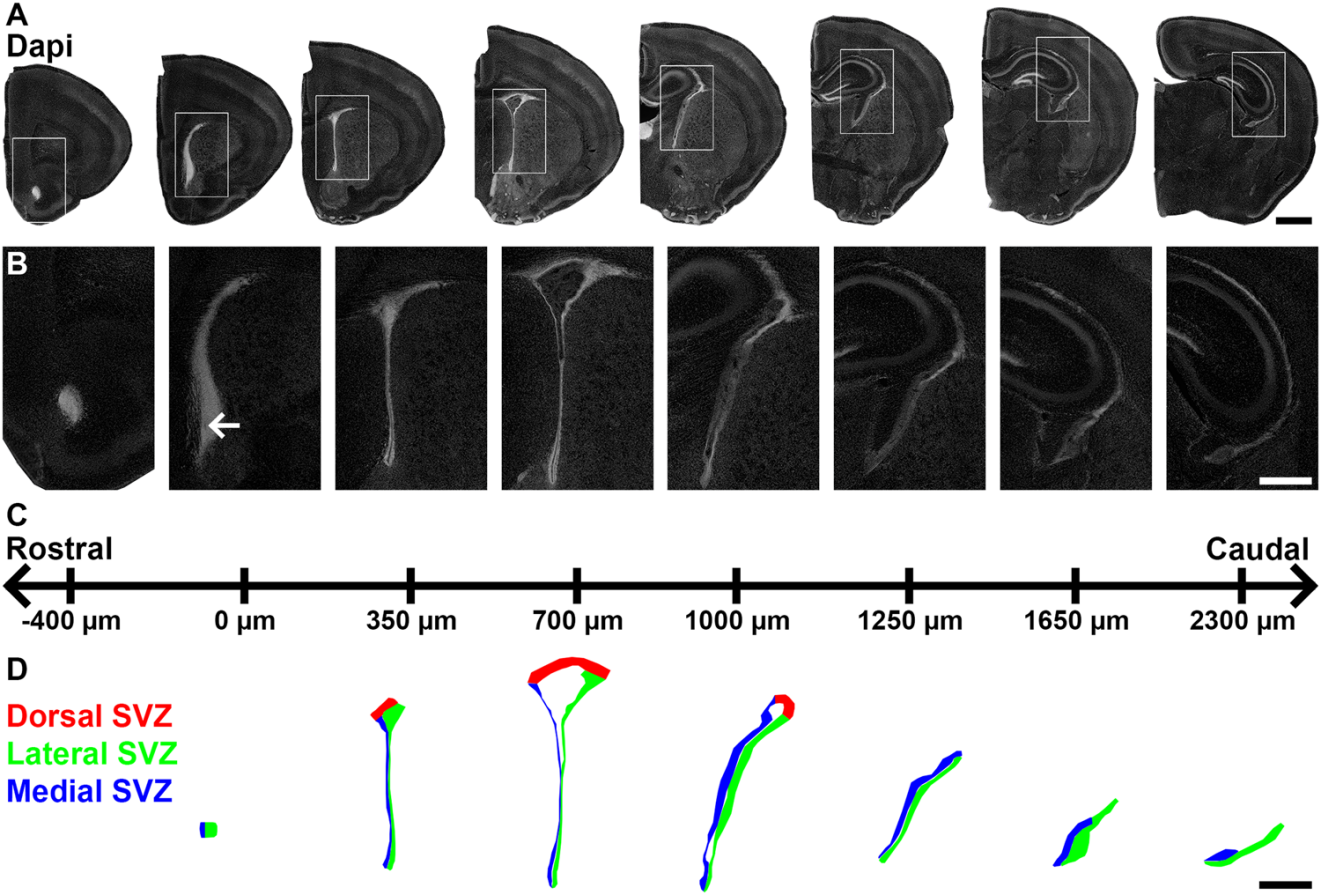
Definition of SVZ microdomains throughout the rostro-caudal LV coordinates. **(A)** Representative mosaics of P10 coronal sections counterstained with Dapi at various rostro-caudal coordinates (indicated in C). **(B)** Higher magnification micrographs showing the rostro-caudal extension of the open LV from its first rostral appearance (arrow) to its more caudal coordinates. **(C)** Coordinates used in this study. The coordinate “0” is defined as the first appearance of the opened LV. **(D)** Schematic representation of the opened LV at various rostro-caudal coordinates. The dorsal (red), lateral (green) and medial (blue) microdomains are indicated. Scale bars: (A) = 1 mm; (B,C) = 500 µm. Abbreviations: SVZ, subventricular zone; LV, lateral ventricle.

The SVZ of the mouse brain is a highly complex and irregular structure, well known for its persisting germinal activity after birth^1,13,14^. It surrounds the LVs, which extends at the postnatal day 10 (P10) approximately 2.5 mm along the forebrain rostro-caudal axis. The SVZ is defined by its dense cellular organization compared to the surrounding tissue, i.e. striatum, corpus callosum, as well as septum that respectively line its lateral, dorsal and medial aspects (Fig. 1A,B).

We first aimed at defining the SVZ from its rostral most aspect corresponding to the appearance of the opened LV (coordinate 0 in Fig. 1C) to its more caudal aspect. Classically, the SVZ is subdivided into 3 microdomains (lateral, dorsal, medial) according to their embryonic origin and respective location around the LV. While these 3 microdomains are contiguous in the rostral most SVZ regions, their definition becomes more ambiguous in regions caudal to the 3^rd^ ventricle. This applies particularly to the dSVZ, which is absent in caudal sections. There, only the lateral and medial walls can be identified based on a dense Dapi counterstaining (Fig. 1D) as well as the presence of numerous Ki67^+^ cells (see below). It is noticeable that the density of cells (detectable by Dapi) declines from rostral to caudal sections. This is particularly apparent in the more caudal sections, where the ventricular walls develop in thicker cell layer of lower cell density (Fig. 1B). Those caudal sections were however included in our analysis in order to directly assess the presence of regions with high versus low germinal activity along the full SVZ, according to the distribution of proliferation and/or progenitor markers (see below).

### “FlashMap” allows rapid analysis of marker expression along the full extent of the SVZ coordinates

**Figure 2:**
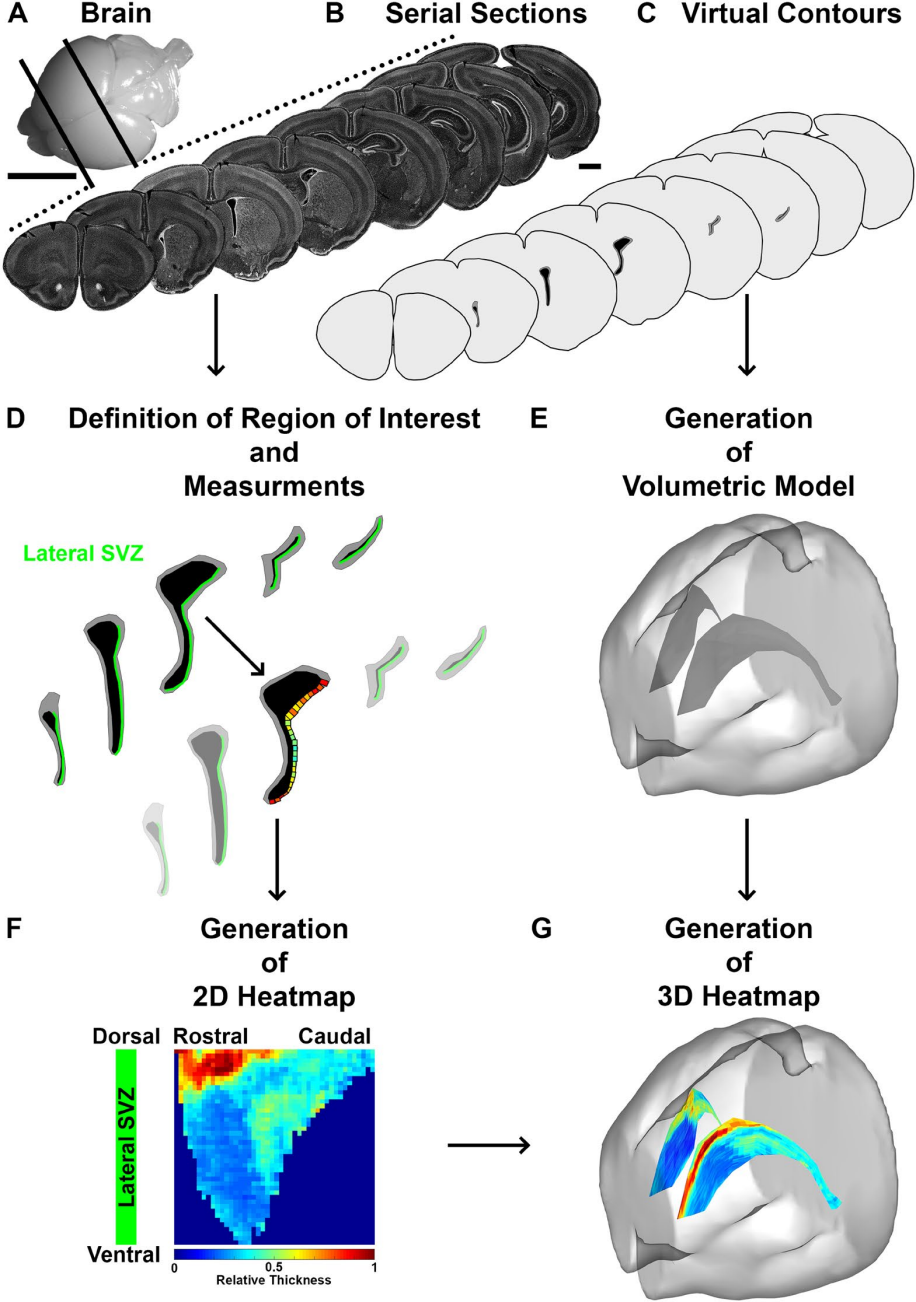
Schematic illustration of the full procedure for sampling and analysis of the SVZ. **(A)** Representative picture of a P10 mouse brain prior to cutting. **(B)** Representative micrographs show a subsampled series of counterstained brain sections (Dapi) covering the full rostro-caudal extent of the LV. Sections are collected/subsampled in series according to the experimental question (here for illustration a subsampling of 1 section out of 12). **(C**,**E)** Schematic illustration of virtual contours of serial brain sections (**C**), which are used for the generation of 3D reconstructions of the brain (bright grey) and LVs (dark grey; **E**). (**D**) Schematic illustration of the definition of the ROI (here lSVZ in green) and the automatic distribution of probes for OD measurements of the investigated marker. Probes width are defined by the user (in this study 50 µm), while the height is calculated by “FlashMap”. (**F**) The software generates automatically 2D heatmaps of SVZ thickness and OD values along the rostro-caudal axis of the ROI (here lSVZ). Values (here SVZ thickness) are represented by a relative colour code (deep blue = 0 to deep red = 1 = highest value). (**G**) Heatmaps can be presented in 3D, by superimposing those onto a 3D reconstruction of the ventricular system (here complete heatmap of SVZ thickness). Scale bars: (A) = 5 mm; (B, C) = 1 mm. Abbreviations: SVZ, subventricular zone; 2D, two dimensional; 3D, three dimensional; OD, optical density; ROI, region of interest; LV, lateral ventricle.

**Figure 3:**
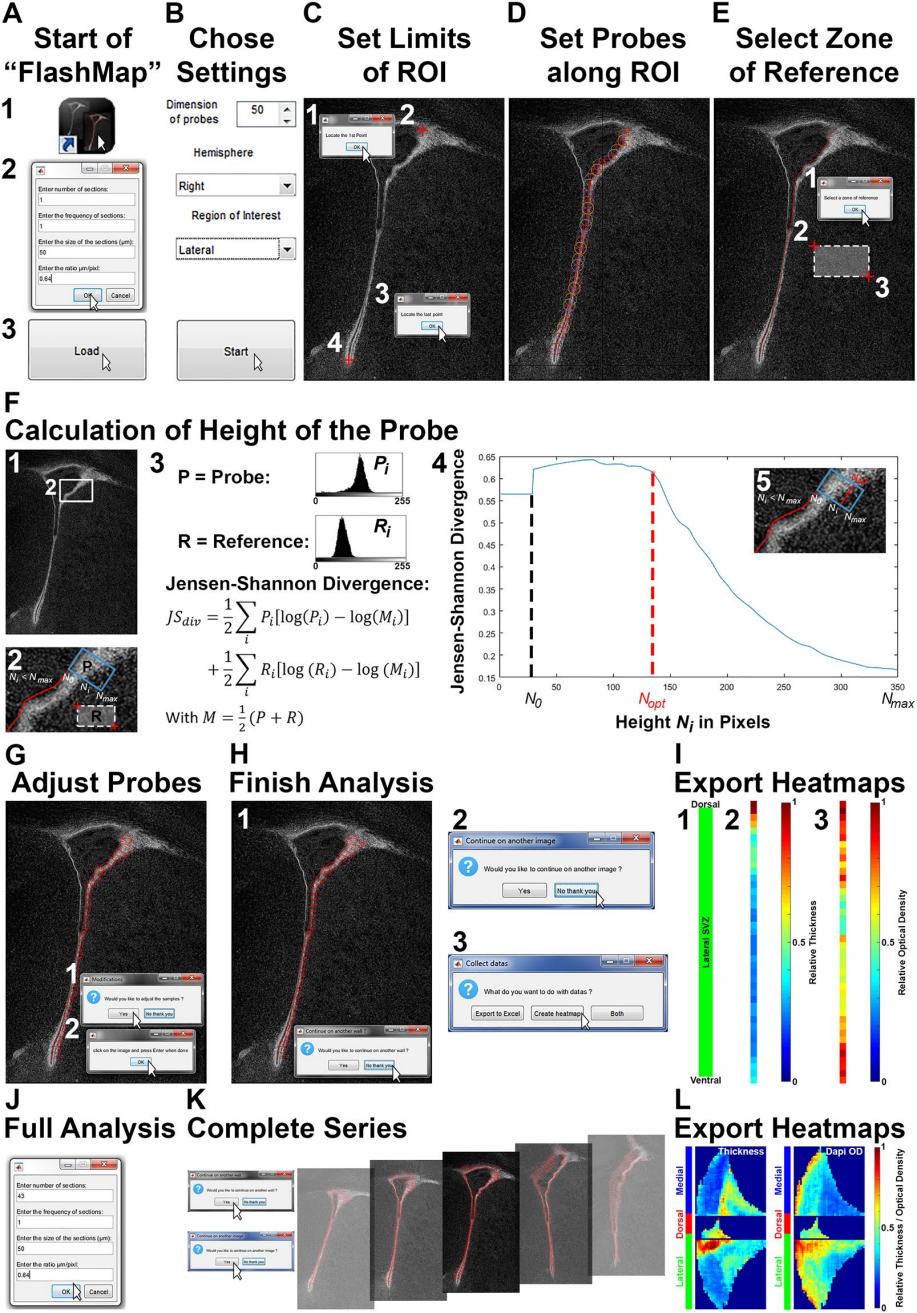
Detailed “FlashMap” walkthrough. **(A)** Start “FlashMap” and load images (1–3). For illustration, we chose to analyse a single microdomain in a single section. Define the thickness of the sections and the µm/pixels ratio (here 50 µm and 0.64 µm/pixel; 2). Load the image (3). **(B)** Define the dimension of the probes (here 50 µm), the hemisphere (here “right” hemisphere) and the ROI (here “lateral” SVZ). **(C)** Define ROI by placing a “start point” (1, 2) and an “end point” (3, 4). **(D)** Draw the internal face of the ROI for automatic distribution of probes. **(E)** Define a reference area in the adjacent tissue (here striatum) by selecting two points in that region (1–3). **(F)** “FlashMap” automatically calculates the height of the probes according to the differential cell densities within the probe (P) and the selected reference region (R; 1, 2). Using *JS*_*div*_ (3) the software estimates the optimal basal height of the probe (*N*_*opt*_; height of the probe in pixels, where the value of the *JS*_*div*_ consistently drops) between the apical and the basal borders *N*_0_ and *N*_*max*_, respectively (4, 5). **(G)** Probes can be adjusted manually. **(H)** Complete the analysis by declining the request to proceed with another ROI (1) and another section (2). Export values (Excel files) and/or heatmaps in TIFF format (3). **(I)** Heatmaps of the lSVZ analysis (here a single section) are orientated with its dorsal end at the top and ventral end at the bottom (1). Heatmaps of the SVZ thickness and cellular density (here Dapi) are colour coded (here using the “jet” preset code) with a scale of relative thickness and OD, respectively (2, 3). **(J,K)** Illustration of an analysis with a complete series of 43 sections. The number of sections to be analysed is defined at the start (step A2). Analysis of multiple walls is achieved by clicking “continue with another wall” (H1). Analysis of multiple sections requires clicking “continue on another image” (H2) until the series is completed (K). **(L)** Full heatmaps are gained as described in (H3) with the same scale bars as described in (I). Orientation of full heatmaps is described in Fig. 4B. Abbreviations: ROI, region of interest; *JS*_*div*_, Jensen-Shannon divergence; SVZ, subventricular zone; lSVZ, lateral subventricular zone; OD, optical density. See also Supplementary Fig. S1.

We developed “FlashMap” to perform a rapid analysis of marker expression in the SVZ along its full rostro-caudal as well as dorso-ventral coordinates. “FlashMap” allows the generation of two-dimensional (2D) heatmaps of relative OD in the least time and tissue consuming way. The reasoning behind “FlashMap” is illustrated in Fig. 2, while a more detailed step-by-step workflow is given in Fig. 3. Brains of the species and age of interest (here, P10 mice) are sectioned and collected in series. For three-dimensional (3D) analysis of gene and protein expression in the SVZ, coronal sections must be preferred (Fig. 2A,B). Sections are immunostained for markers of interest. To reduce time and tissue consumption, series may be subsampled. The subsampling factor will depend on the abundance of the marker of interest as well as of its distribution. Thus, classical rules used in stereology apply: i.e. less abundant markers showing heterogeneous distribution require low subsampling, for maximal resolution. After definition of the region of interest (ROI; here lSVZ in green; Fig. 2D), “FlashMap” defines automatically the thickness of the SVZ based on the OD of the Dapi counterstaining (as described later in more detail). The software distributes probes of a defined width and automatically adapts their height (corresponding to thickness of the SVZ). The OD of the markers of interest is then measured within each probe (Fig. 2D). These ODs are then represented in a colour code of relative OD (from 0 = deep blue to 1 representing the highest value = deep red). A 2D heatmap is generated, which represents the SVZ thickness or marker distribution along the ROI. Analysis of multiple sections allows representing the full rostro-caudal extend of the ROI (Fig. 2F). For 3D representation, the contours of a series of sections (Fig. 2C) are used to generate a 3D mesh of the LV (Fig. 2E) using Neurolucida 360 (MBF Bioscience). 2D heatmaps are then superimposed onto these 3D volumes by using the open access software Blender (www.blender.org; Fig. 2G; see Supplementary Blender file “Template 3D Heatmap”).

### Step-by-step guidelines for installation and optimal use of “FlashMap”

While installing the software (see Supplementary Software “FlashMap 1.0”), a shortcut to “FlashMap” should be placed on the desktop (Fig. 3A1) as well as all the images which will be analysed. After starting “FlashMap”, a first window appears where the name of the study as well as green and red channels need to be indicated. Following this step a second window opens for guiding the user to define the settings of the analysis to be performed. For illustrative purposes, we chose to analyse one microdomain in one section (number of sections: 1; frequency of sections: 1). The thickness of the sections (here 50 µm) and the µm/pixel ratio also need to be defined (here 0.64 µm/pixel; Fig. 3A2). Thereafter, the image is loaded by clicking the “Load” icon (Fig. 3A3). The dimension of the probes (width in µm) are chosen as well as the hemisphere (left; right) and the ROI (medial; lateral; dorsal) to be analysed (here 50 µm; right; lateral; Fig. 3B). The beginning and end of the ROI are defined by accepting the request and clicking (left or right) on the image at the appropriate position (i.e. clicks 1 and 2, 3 and 4, respectively in Fig. 3C). Maximal accuracy can be achieved using the zoom and navigation icons in the tool bar. The internal side of the ROI is traced for automatic positioning of the probes. The lSVZ and medial SVZ (mSVZ) must be traced from dorsal to ventral, while the dSVZ must be traced from lateral to medial regardless of the hemisphere. Clicking on the “end point” of the ROI finishes the tracing and can therefore be used as an escape, if a mistake is made (Fig. 3D). A “reference region” is placed within the parenchyma, immediately adjacent to the SVZ (here in the striatum; Fig. 3E), to enable “FlashMap” to automatically calculate the height of the probes. Probes height is defined by using the Jensen-Shannon divergence^15^ (*JS*_*div*_) to accurately define the drop in OD values for the Dapi nuclear counterstaining occurring between the SVZ and the adjacent tissue (grey values in the “probe region” (*P*) and “reference region” (*R*; Fig. 3F1,F2)). In the *JS*_*div*_ the normalized values *P*_*i*_ and *R*_*i*_ represent the intensity versus the frequency of grey values in the “probe region” and the “reference region” for the height *N*_*i*_ of the probe. The *JS*_*div*_ measures the dissimilarity between the grey value distribution between them (Fig. 3F2,F3):

$$J{S}_{div}=\frac{1}{2}\sum _{i}{P}_{i}[\mathrm{log}({P}_{i})-\,\mathrm{log}({M}_{i})]+\frac{1}{2}\sum _{i}{R}_{i}[\mathrm{log}({R}_{i})-\,\mathrm{log}({M}_{i})]$$

With

$$M=\frac{1}{2}(P+R)$$

*N*_0_ and *N*_*max*_ are empirically fixed values and “FlashMap” automatically estimates the optimal height (*N*_*opt*_) for every single probe. *N*_*opt*_ is defined as the height at which the *JS*_*div*_ value continuously drops as the grey value distributions of *P*_*i*_ and *R*_*i*_ become identical (Fig. 3F4,F5). Importantly, the estimation of *N*_*opt*_ starts at *N*_*0*_ larger than the real apical end of the probe (0 pixel) at 30 pixels, as *JS*_*div*_ exhibits spurious variations for very small values. We further tested if the calculated height of the probes differ by selection of different “reference regions”. While the *JS*_*div*_ values were slightly different, the calculated positions *N*_*i*_ for *N*_*opt*_ appeared to be very consistent. Thus, a precise positioning of the “reference region” is not necessary, as it does not affect optimal detection of the SVZ thickness (for illustration see Supplementary Fig. S1). Importantly, rare errors in the automatic estimations of probe heights can be corrected manually. First, the probe is selected by clicking close to its apical side. The corrected height is then chosen by clicking at the optimal position at its basal side (Fig. 3G). The analysis is ended by declining the invitation to analyse other SVZ walls (1) and another image (2). OD measurements can then be exported as raw values in an Excel file and/or as a heatmap (3). It is strongly recommended to always export raw values for accurate interpretation of heatmaps (Fig. 3H). Indeed, heatmaps provide a spatial representation of marker expression, but do not give information on their level of expression (e.g. amplitude between high and low expression values). The heatmaps we produced in this illustration are orientated with the dorsal end of the lSVZ at the top and the ventral end at the bottom (1). Here the thickness of the lSVZ is represented (2) as well as the density of cells along the lSVZ, based on OD measurements of the nuclear counterstain Dapi (3). The heatmaps represent the relative thickness/OD, which are colour coded (here using the preset “jet” color code) from 0 (deep blue) to the maximal measured OD value (normalized to 1; deep red; Fig. 3I). Different options for colour coding of OD values are available in “FlashMap” and can be chosen prior to the heatmap export.

A similar procedure will be used to analyse multiple sections. The total number of sections is defined at the start (step A2, here 43 sections) before loading the first image. Subsampling can be done, as described below, but requires defining the “frequency of sections” (here “1”, as all sections were processed) in the same window (Fig. 3J). The full SVZ of the series can then be analysed as illustrated in Fig. 3K. Multiple LV walls are sampled on the same series of images by choosing “continue with another wall” (Fig. 3H1). After defining all walls within a section, the option “continue on another image” is selected (Fig. 3H2) to load the next image. A list of already processed images appears to help the user in keeping track with the procedure. After analysis of all the sections the heatmaps can be generated as described previously (Fig. 3H3). This results in complete heatmaps (here thickness and cellular density of the 3 SVZ walls throughout its full rostro-caudal coordinates; Fig. 3L).

### “FlashMap” highlights regions of maximal germinal activity in the postnatal SVZ

**Figure 4:**
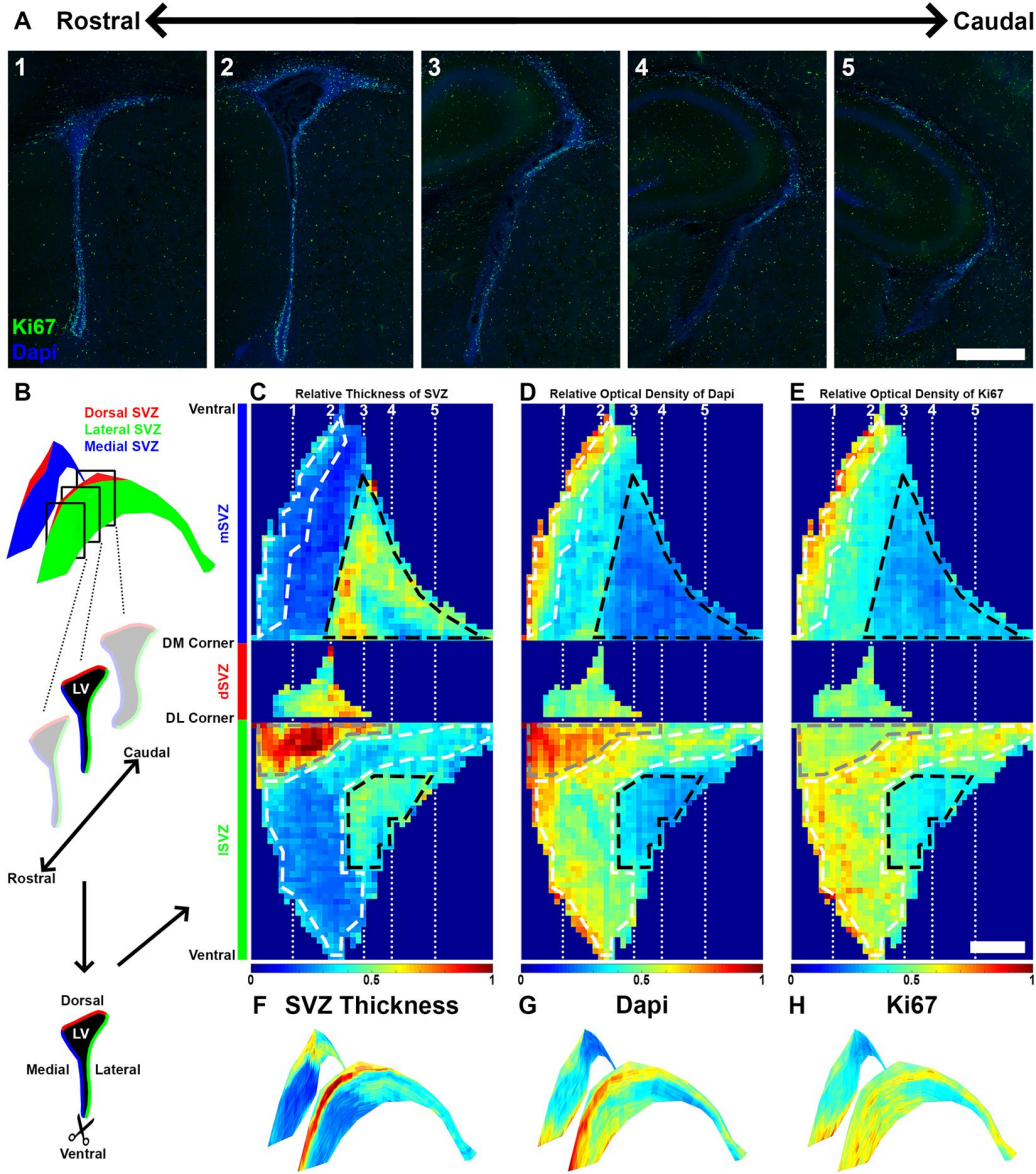
“FlashMap” reveals major differences in SVZ thickness, cellular density and germinal activity throughout the SVZ. **(A)** Representative micrographs of the proliferation marker Ki67 (green) at distinct rostro-caudal SVZ coordinates (Dapi, blue). **(B)** Schematic representation of sections and heatmaps orientations. The LV is cut open (scissors) at its most ventral tip between the mSVZ (blue) and the lSVZ (green). This allows unfolding the three SVZ walls for optimal 2D heatmap representations (see **C**–**E**). **(C–E)** Heatmaps of the relative thickness of the SVZ (**C**) and of relative ODs for Dapi (**D**) and Ki67 (**E**) along the full rostro-caudal extent of the LV (left = rostral; right = caudal). White dashed boxes underline regions of maximal germinal activity, while black dashed boxes underline regions of minimal germinal activity. Note the differences existing between these regions in term of SVZ thickness and cellular density. The location of the RMS is highlighted with a grey dashed box. The heatmap colour code goes from 0 (deep blue) to maximal detected thickness/OD 1 (deep red). **(F–H)** 3D projections on the LV of heatmaps for relative SVZ thickness (**F**) and relative ODs for Dapi (**G**) and Ki67 (**H**). Scale bars: (A,E) = 500 µm. Abbreviations: SVZ, subventricular zone; lSVZ, lateral subventricular zone; dSVZ, dorsal subventricular zone; mSVZ, medial subventricular zone; LV, lateral ventricle; DM corner, dorso-medial corner; DL corner, dorso-lateral corner.

To test “FlashMap”, we first aimed at sampling the full SVZ for regions of maximal germinal activity. The SVZ thickness and cellular density as well as the distribution of proliferative cells were mapped throughout the rostro-caudal extend of the three SVZ walls (Fig. 4A). As a marker of proliferation, we chose Ki67, which is expressed by actively cycling cells^16^.

For optimal heatmap orientation, we virtually opened the LV at its ventral tip along the full rostro-caudal extent, in order to “unfold” the ventricle and represent the medial, dorsal and lateral walls on top of each other in a 2D heatmap (Fig. 4B). We first generated heatmaps for the relative thickness and cellular density throughout the SVZ, based on the Dapi counterstaining (Fig. 4C,D). Analysis of the SVZ thickness revealed the existence of a “hotspot” at the junction between the lSVZ and the dSVZ. This region corresponds to the accumulation of neuroblasts before they engage into rostral migration through the RMS. In this region the increased SVZ thickness correlates with an increased cell density (Fig. 4C,D; grey dashed boxes). Heatmaps also reveal a gradual increase in SVZ thickness in more caudal regions of both the lSVZ and mSVZ. Interestingly, this increase correlated with a decrease in cell density, suggesting a gradual cell dispersion in these more caudal regions, which may correlate with a loss of germinal activity. We then generated a heatmap for the density of proliferative cells (i.e. Ki67^+^ cells; Fig. 4E). The highest germinal activity was detected in a “banana shaped area” along the rostro-caudal extent of the lSVZ as well as in the most ventral and rostral regions of the mSVZ. Interestingly, comparison of the thickness of the walls, cellular density and density of Ki67^+^ cells revealed that the regions with highest germinal activity also appear to be thin, but very dense (Fig. 4C–E; white dashed boxes). In contrast, regions of lowest germinal activity were thicker and presented a lower cellular density (Fig. 4C–E; black dashed boxes). For a more intuitive representation, heatmaps were superimposed on 3D templates of the LVs using Blender (Fig. 4F–H), which also allows the generation of 360° 3D movies (see Supplementary Blender file “Template 3D Heatmap”, and Supplementary Movie “3D SVZ Thickness”).

### Subsampling results in a minimal loss of spatial information

**Figure 5:**
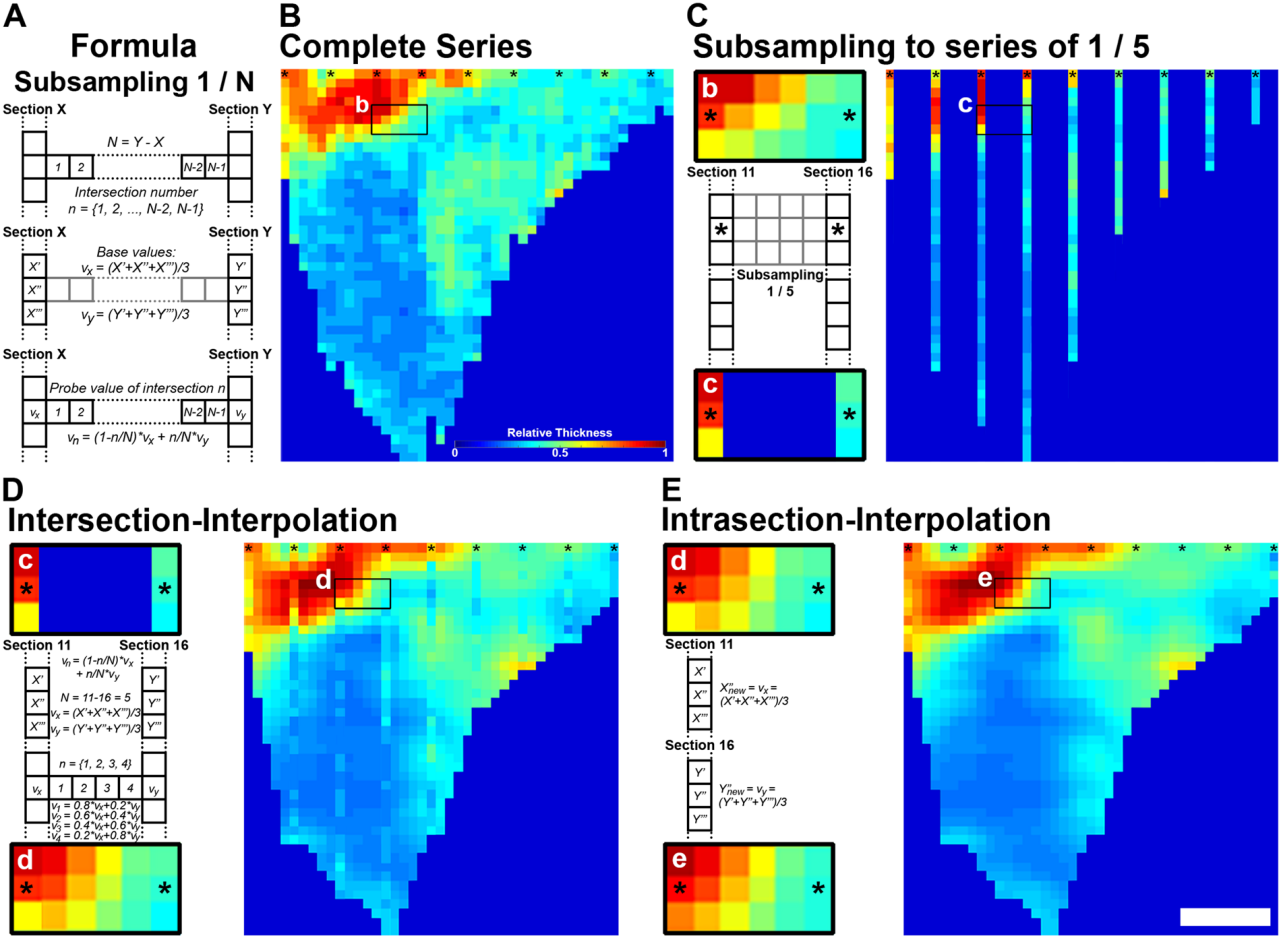
Interpolation of heatmaps after 1/5 subsampling. **(A)** Formula for intersection-interpolation. **(B)** Heatmap representing the thickness of the lateral wall in a complete series of sections. **(C–E)** Heatmaps representing values obtained for a 1/5 subsampling, without interpolation (**C**), following “intersection-interpolation” (**D**) and “intrasection-interpolation” (**E**). Formula are presented on the left as well as a cropping of a region of reference for all heatmaps. Scale bar: (**B**) = colour coded relative thickness; (**E**) = 500 µm. See also Supplementary Figs S2–S4.

In order to reduce the time and amount of tissue required to perform an analysis, we implemented subsampling to “FlashMap” (i.e. to perform an analysis of 1 section out of *N*). The applied calculations for interpolating missing values, follows the concept of linear interpolation. Values between two analysed sections (X; Y) were calculated by using the following formula (Fig. 5A):

$${v}_{n}=(1-\frac{n}{N})\ast {v}_{x}+\frac{n}{N}\ast {v}_{y}$$

in which *N* stands for the degree of subsampling and *n* for the intersection number between real sections X in rostral and Y in caudal direction:

$$\begin{array}{ccc}N & = & degree\,of\,subsampling\\ n & = & \{1,\,2,\,\ldots ,\,N-2,\,N-1\}\end{array}$$

The smoothening of the values interpolation is applied row- by- row as follows. First, “base values” (*v*_*x*_; *v*_*y*_) of the two adjacent real sections are calculated. This is achieved by averaging the real values (*X″*; *Y″* ) with its neighbours (*X′*, *X″′*; *Y′*, *Y″′* ) for every row and section separately:

$$\begin{array}{ccc}{v}_{x} & = & \frac{X^{\prime} +X^{\prime\prime} +X^{\prime\prime} \,^{\prime} }{3}\\ {v}_{y} & = & \frac{Y^{\prime} +Y^{\prime\prime} +Y^{\prime\prime} \,^{\prime} }{3}\end{array}$$

For probes located at the border of the ROI, a single adjacent measured value was available and therefore used:

$$\begin{array}{ccc}{v}_{x} & = & \frac{X^{\prime} \,{\rm{or}}\,X^{\prime\prime} \,^{\prime} +X^{\prime\prime} }{2}\\ {v}_{y} & = & \frac{Y^{\prime} \,{\rm{or}}\,Y^{\prime\prime} \,^{\prime} +Y^{\prime\prime} }{2}\end{array}$$

All the necessary steps of a full interpolation of the SVZ thickness in the lSVZ are illustrated in Fig. 5E. For illustrative purposes, we generated a complete heatmap (Fig. 5B) and one by analysing every 5^th^ section (i.e. 1/5 subsampling; Fig. 5C). We then interpolated missing values in two steps. For the first step, the “intersection-interpolation” (Fig. 5D), the general formula is used as described above with:

$$\begin{array}{c}N=5\\ n=\{1,\,2,\,3,\,4\}\end{array}$$

Its integration into the general formula reads as follow:

$${v}_{1,2,3,4}=(1-\frac{{n}_{1,2,3,4}}{5})\ast {v}_{x}+\frac{{n}_{1,2,3,4}}{5}\ast {v}_{y}$$

Weighting of the influence of adjacent sections in the series to calculate values for the four missing sections (*v*_1_, *v*_2_, *v*_3_, *v*_4_) reads as follow:

$$\begin{array}{c}{v}_{1}=0.8\ast {v}_{x}+0.2\ast {v}_{y}\\ {v}_{2}=0.6\ast {v}_{x}+0.4\ast {v}_{y}\\ {v}_{3}=0.4\ast {v}_{x}+0.6\ast {v}_{y}\\ {v}_{4}=0.2\ast {v}_{x}+0.8\ast {v}_{y}\end{array}$$

Comparison of the heatmaps generated following analysis of a complete series of sections or a 1/5 subsampling (compare Fig. 5B,D) highlight the accuracy of the “intersection-interpolation”. A second step of “intrasection-interpolation” allows a further refinement of the heatmap (Fig. 5E), while preserving the accuracy of the mapping.

Next, we systematically assessed the degree of subsampling allowing the production of a faithful map, as obtained with a complete series of sections. We repeated all analysis with sub-samplings ranging from 1/2 to 1/6. Interestingly, all previously described regions (germinal regions, non-germinal regions, RMS; Fig. 4C–E) could be identified in all heatmaps, regardless of the degree of subsampling or the section number with which the analyses were started (see Supplementary Figs S2–S4).

### “FlashMap” allows a rapid analysis of SVZ transcriptional heterogeneity

**Figure 6:**
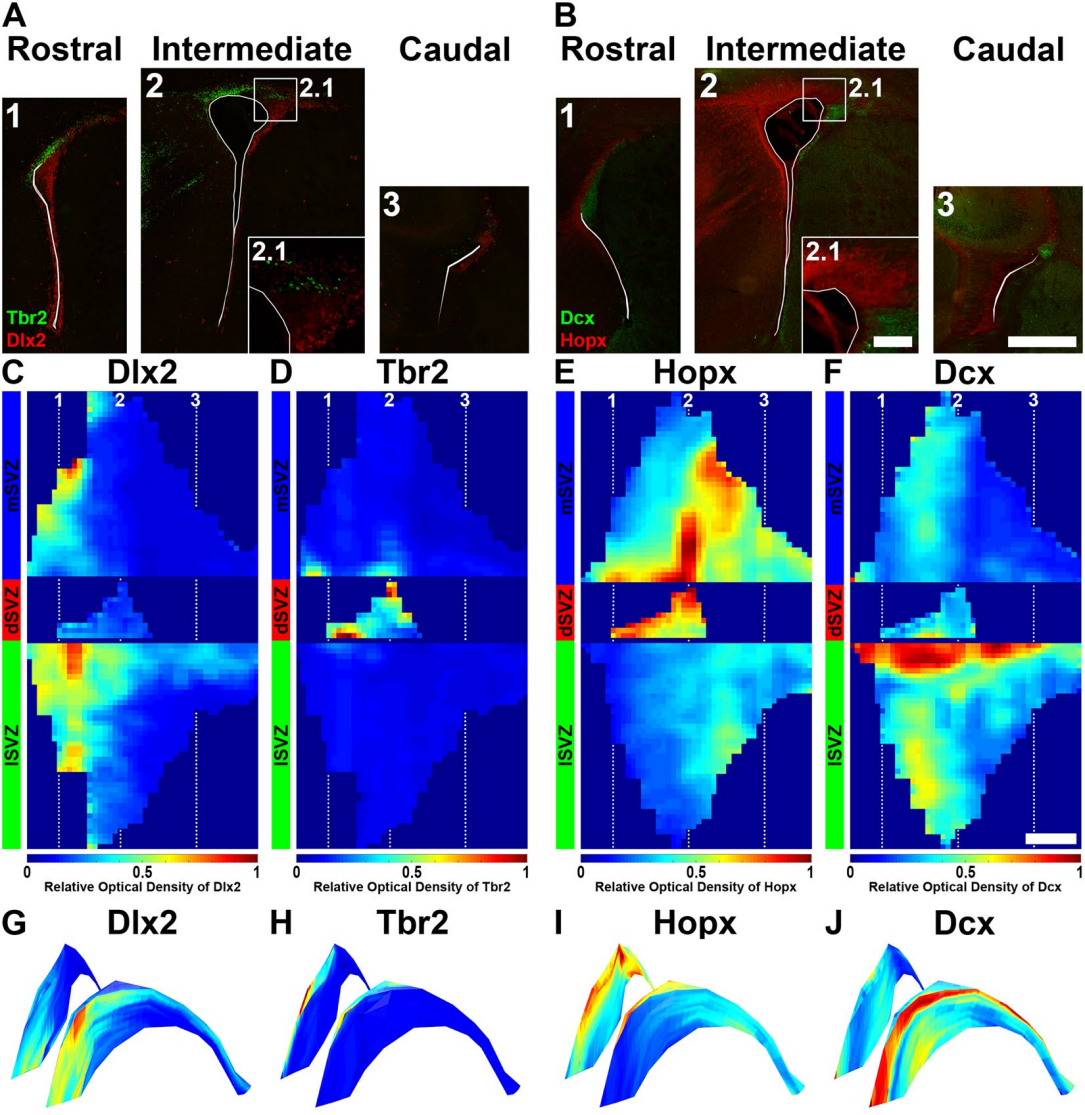
Illustration of “FlashMap” analysis with four regionally expressed markers. **(A,B)** Representative micrographs of Dlx2, Tbr2 (**A**), Hopx and Dcx (**B**) at rostral (A1,B1), intermediate (A2,B2) and caudal coordinates (A3,B3) along the rostro-caudal axis of the ventricular system. **(C–F)** 2D expression heatmaps obtained for Dlx2 (**C**), Tbr2 (**D**), Hopx (**E**) and Dcx (**F**) along the full extent of the SVZ are represented in a colour code of relative OD. **(G–J)** 2D heatmaps of Dlx2 (**G**), Tbr2 (H), Hopx (**I**) and Dcx (**J**) are superimposed in 3D LVs. Scale bars: (B2.1) = 100 µm; (B3,F) = 500 µm. Abbreviations: 2D, two-dimensional; 3D, three-dimensional; lSVZ, lateral subventricular zone; dSVZ, dorsal subventricular zone; mSVZ, medial subventricular zone.

Next, we used “FlashMap” to generate full heatmaps of selected markers, known to be heterogeneously expressed in the postnatal SVZ. First, we chose the GABAergic and glutamatergic progenitor markers Dlx2 and Tbr2 (Fig. 6A). Those two transcription factors show a preferential expression in the lSVZ and the dSVZ, respectively^8,17,18^.We further analysed the expression pattern of Hopx and Dcx. Hopx is a heterogeneously distributed transcriptional regulator, which recently gained increasing attention as a marker for outer radial glia cells in humans^19,20^. Dcx is a marker for immature neurons^21^ and therefore labels neuroblasts which accumulate in specific regions within the SVZ^22^ (Fig. 6B).

Remarkably, Dlx2 was detected in the lSVZ and the mSVZ in the regions we previously identified as zones of high germinal activity (Fig. 4C–E; white dashed boxes). Highest Dlx2 expression was observed in the rostral parts of the lSVZ as well as in the ventral tip of the mSVZ in rostral sections. A marked expression was also detected in the dorsal aspect of the lSVZ, corresponding to the RMS, in line with the persistent Dlx2 expression in migrating neuroblasts^18^. In contrast, Dlx2 was consistently absent from the dSVZ and more caudal regions of the lSVZ and mSVZ (Fig. 6C,G). This expression pattern contrasts to the one obtained for Tbr2, which showed an exclusive expression in the dSVZ and was consistently absent from the lSVZ and mSVZ (Fig. 6D,H). Those findings are in line with previous reports^8,17,18^ and further validate “FlashMap” as a rapid and accurate mapping method.

“FlashMap” revealed a strikingly different pattern for Hopx expression. Hopx was found to be high in the dSVZ and in the caudal regions of the mSVZ. Some level of expression could also be detected in the caudal regions of the lSVZ, where only few Ki67^+^ cells are observed. Note the high accuracy of “FlashMap” in detecting and representing the expression gradient of Hopx in the dSVZ as described elsewhere^23^ (Fig. 6E,I). It is noticeable that this pattern of expression is the opposite of the one obtained for Dcx. As revealed by the “FlashMap” analysis, this migrating neuroblast marker is enriched in the dorsal part of the lSVZ, where the RMS is located. A second smaller “hotspot” was also observed in the ventral parts of the lSVZ in rostral sections (Fig. 6F,J).

### “FlashMap” allows averaging animals for quantitative analyses

**Figure 7:**
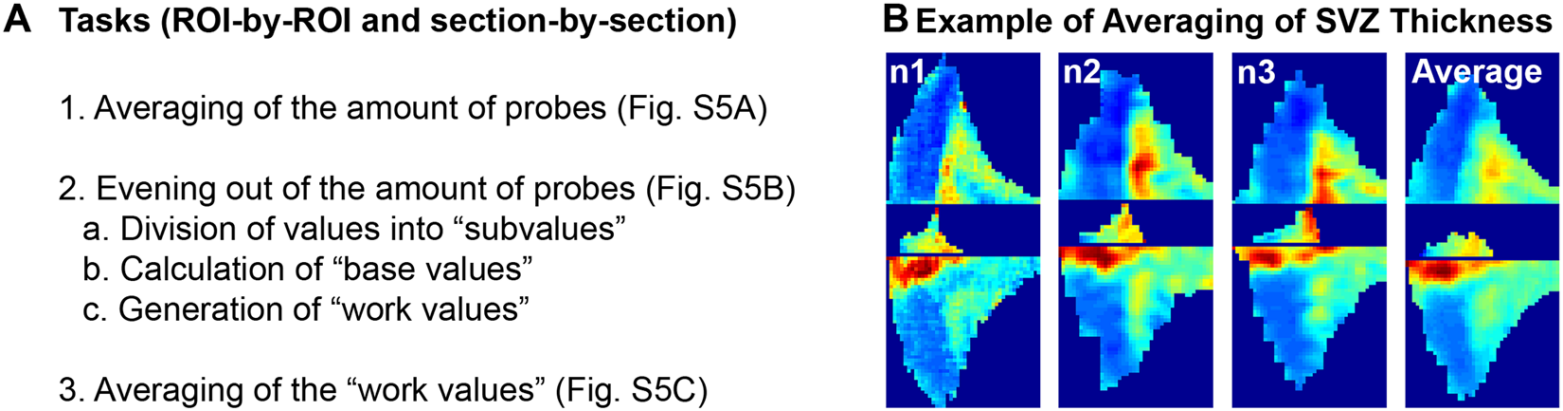
Averaging of different datasets using “FlashMap”. **(A)** List of steps performed by “FlashMap” for averaging datasets. All steps are performed ROI-by-ROI and section-by-section. In a first step the amount of probes is averaged (1), which allows to even out the amount of probes by dividing the values into ‘subvalues” (2a) to calculate “base values” (2b) and generate “work values” (2c). Finally, the new “work values” are averaged by “FlashMap” (3). **(B)** Heatmaps represent the relative SVZ thickness of 3 independent analyses (n1, n2, n3) as well as an averaged heatmap generated by “FlashMap”. Abbreviation: ROI, region of interest; SVZ, subventricular zone. For a more detailed illustration see also Supplementary Fig. S5.

Analysis of a cohort of animals may be of interest to reduce inter-individual variability or compare experimental groups. This additional feature was added to “FlashMap”. The necessary steps to average data obtained from different animals are described in Fig. 7A and a more detailed illustration is provided in Fig. S5. The following steps are automatically performed by “FlashMap”, when Excel files corresponding to distinct animals are uploaded. Note that if 1 is set as the amount of animals, “FlashMap” will ask to upload the Excel files corresponding to the 3 channels of this animal (1 for SVZ thickness and 2 for the markers of interest) and generate the 3 corresponding heatmaps. If the amount of animals is set as larger than 1, “FlashMap” will ask for the Excel files concerning one specific channel of all independent analyses from animals to be averaged. Averaging is performed ROI-by-ROI and section-by-section as follows: First, the number of probes Y_n_ of the different individuals (in our example n = {1, 2, 3} with the amount of probes = {1, 4, 5} in the illustration in the Supplementary Fig. S5) is averaged to obtain the amount of final probes, i.e. X (here 3), to represent (Fig. 7A1; see also Supplementary Fig. S5A). Then, all values Y_n_ are divided by X to obtain Y_n_ * X “subvalues”. The amount of Y_n_ of these “subvalues” are then averaged resulting in X “base values”, which are further multiplied by X. This calculation allows defining the same amount of probes (X) in a given section with corresponding “work values” for all animals (Fig. 7A2; see also Supplementary Fig. S5B). Finally, “work values” from individual animals are processed to obtain an averaged representation of SVZ thickness or ODs distribution (Fig. 7A3; see also Supplementary Fig. S5C). As an illustration of this approach, we averaged 3 datasets of SVZ thickness from independent analyses (Fig. 7B). The resulting map generated by “FlashMap” accurately reflects the spatial organisation of the SVZ, while integrating inter-individual variability.

## Discussion

Our results establish “FlashMap” as a useful tool for a rapid spatial analysis of marker expression along both the rostro-caudal and dorso-ventral coordinates of the postnatal SVZ. Analysis of select markers highlight marked spatial differences along the SVZ in term of germinal activity and distribution of progenitors of distinct lineages. This software will prove to be useful for systematic 3D analysis and representation of marker expression in the SVZ.

The SVZ is an ill-defined region of the postnatal mouse forebrain. The SVZ is particularly thin and tortious, and our results further demonstrate that its cellular density greatly varies along its dorso-ventral and rostro-caudal dimensions. To perform the current study, we defined the dorsal, lateral and medial walls of the opened LV along its full rostro-caudal coordinates. A more accurate subdivision of the three walls may be achieved by using tissues from transgenic mice expressing permanent markers in pallial and subpallial regions of the developing forebrain^24^. Although the approach used in the current study remains arbitrary, it allowed detecting significant differences in the thickness, cell density and proliferation occurring at different coordinates of the postnatal SVZ. Our results confirm previous results obtained for the distribution of proliferating cells (Ki67^+^) in the lSVZ^25^ and refine them significantly by providing a more complete and precise map. In addition to the rostro-caudal gradient observed for the distribution of proliferative cells, which is in agreement to this previous report, we found a lower germinal activity in the dorsal part of the lSVZ, where the RMS is located. We also provide a more detailed mapping of the density of proliferative cells in more caudal regions, where a large region of low germinal activity is clearly visible. The differences existing between our maps and those generated by Falcao *et al*., may be at least partially explained by differences in the age of the animals (P10 vs. P70). A systematic mapping at different ages using “FlashMap” would be of interest to better describe spatial changes in germinal activity that occur over a lifetime. Such an analysis could also include embryonic time points to investigate in more details the spatial and temporal aspects of astrogenesis and oligodendrogenesis (reviewed in Reemst *et al*.^26^). This would be particularly interesting for oligodendrogenesis, as it occurs in different waves originating from different regions of the VZ and SVZ^10^.

Interestingly, the regions of “lowest germinal activity” highlighted by “FlashMap” in the SVZ microdomains shared some key characteristics: i.e. a thick wall with a low cell density. These observations highlight the importance of the cellular microenvironment in the persistence of a germinal activity. This is in agreement with previous studies demonstrating the importance of a highly organized cellular niche (also named neurogenic niche) in the maintenance of both proliferation and neurogenesis (e.g. Ponti *et al*.^27^). Further, we demonstrated that the contribution of the ventral most part of the mSVZ might be underestimated in its participation to the germinal activity observed in the postnatal SVZ. While progenies from the dorsal proliferating cells most likely migrate through the RMS into the OB, some progenies of the ventral population of the lSVZ and mSVZ might migrate away in a more ventrally located stream as suggested elsewhere^22^.

Further, we confirmed in this study the value and reliability of “FlashMap”, by analysing markers known to be regionally expressed, i.e. the transcription factors Dlx2 and Tbr2. The produced heatmaps reflect accurately the prior description of their distribution^8,17,18^. In addition, comparison of Hopx and Dcx highlights the existence of two domains: one presenting a high germinal activity in which neuroblasts are numerous and one presenting a low level of germinal activity that is free of neuroblasts. These observations question the role of Hopx in shaping SVZ germinal activity, and motivated additional experiments aimed at investigating its role in NSCs activity and specification, as recently published^23^.

Several aspects of “FlashMap” have been optimized to make it both user friendly, accurate and rapid. First, we used the *JS*_*div*_ to automatically adapt the probes to the varying thickness of the SVZ. *JS*_*div*_ has been extensively used for multiple types of image processing and analysis, such as the analysis of edge detection^28^, the characterization of the compositional complexity^29^, the tracking of moving root sections in a stack for tracing out the 3D root architecture^30^ and others. Second, we performed extensive subsampling in order to demonstrate that accurate maps can be obtained with an incomplete (but homogeneous) series of sections, such as those classically used in stereology. This allows a reduction of both the time and tissue required to generate an expression heatmap. Indeed, a series of one section out of five reduces the time required for performing the staining, mounting and imaging to 20%. In other words, subsampling allows the parallel analysis of several markers in approximately the same time than required for a single marker in a full series. Third, “FlashMap” is able to average data obtained by independent analyses. This allows to efficiently compare groups of animals, which received different treatments, have a divergent genetic background or were kept under different experimental conditions. Thus, “FlashMap” could facilitate analysis of tissue organization, proliferation and apoptosis in various physiological and pathological conditions. It could also be applied to other brain regions (germinal or non-germinal). For such approaches, some small modifications in the detection mechanism of the probes height may be necessary.

Altogether, this work illustrates the benefits to investigate the spatial organization of germinal regions in details, to guide research in genes involved in NSCs self-renewal and fate specification. It proposes “FlashMap” as an open source, user-friendly and flexible software to perform such, otherwise time consuming analyses.

## Materials and Methods

### Animals and ethics

All animal experimentation procedures were performed according to European requirements 2010/63/UE and have been approved by the Animal Care and Use Committee CELYNE (APAFIS #187 & 188). OF1 mice (Charles River, France; n = 3) were sacrificed at P10 by an intraperitoneal overdose of pentobarbital followed by transcardial perfusion with Ringer Lactate solution and 4% paraformaldehyde (PFA) dissolved in 0.1 M phosphate buffer (PB; pH 7.4). Tissues were postfixed for 48 hrs in 4% PFA at 4 °C.

### Tissue processing and immunohistochemistry

Brains (Fig. 1A) were cut into 50 µm thick sections using a vibratome (VT1000 S; Leica; Wetzlar; Germany) and LV containing sections were serially collected in series of 6. A subsampling of up to 1/6 (i.e. 1 section out of 6, corresponding to 50 µm sections, 250 µm apart from each other) was used to ensure homogeneous sampling of the ROI, while minimising the number of sections analysed, according to stereological standards. Antigen retrieval was performed for Ki67, Tbr2 and Dlx2 immunostainings by incubating sections for 20 minutes in citrate buffer (pH 6) at 80 °C, cooling for 20 minutes at room temperature, followed by extensive washings in 0.1 M PB. Blocking was performed for 2 hrs in TNB buffer (0.05% Casein; 0.25% Bovine Serum Albumin; 0.25% TopBlock in 0.1 M PB) with 0.4% triton-X (TNB-Tx). Incubation with primary antibodies in TNB-Tx was done overnight at 4 °C. The following primary antibodies were used: Rabbit anti-Ki67 (1:500; MM France Microm Microtech; RM-9106-S1); Rabbit anti-Tbr2 (1:1000; Abcam; ab23345); Guinea pig anti-Dlx2 (1:5000; kind gift of Kazuaki Yoshikawa^31^); Rabbit anti-Hopx (1:400; Santa Cruz; sc-30216); Goat anti-Dcx (1:500; Santa Cruz; sc8066). Sections were extensively washed in 0.1 M PB with 0.4% triton-X (PB-Tx) and incubated for 2 hrs at room temperature with suitable secondary antibodies (Alexafluor 488, 555 or 647; 1:500; Life Technologies). After washing (0.1 M PB) sections were counterstained with Dapi (1:500; Life Technologies; D1306) for 30 minutes for optimal detection of SVZ borders by “FlashMap”. Ki67 staining was performed on a complete series of sections, while a subsampling of 1/3 was used for Tbr2, Dlx2, Hopx and Dcx stainings as well as for generating the 3D brain and ventricular system models.

### Image acquisition and 3D reconstruction

Acquisition of images was performed with a Leica DM5500 epifluorescent microscope (Leica Microsystems GmbH, Wetzlar, Germany). Whole brain mosaics were acquired at 4x (HC PL FLUOTAR; N.A. 0.13) for representative overview pictures and 3D reconstruction, at 10x (HCX PL FLUOTAR; N.A. 0.30) for OD analysis and assembled using the LAS X software (Leica Microsystems GmbH, Wetzlar, Germany). For accurate OD analysis, settings were carefully chosen to avoid signal saturation and were maintained throughout the acquisition. Image mosaics were exported in TIFF format (.tif) for further analysis.

A Dapi series of 1/3 sections was used for generating the 3D brain and ventricular system models. Acquired mosaics were orientated using Photoshop (CS4). Contours of the brain and the ventricular system were drawn on individual sections using Neurolucida 360 (MBF Bioscience) to generate a 3D mesh. Meshes were saved in “obj” format and exported to the open-source 3D computer graphics software Blender (www.blender.org) for further editing. For optimal superimposition of 2D heatmaps onto the 3D ventricular system model, the three SVZ walls (medial, dorsal, lateral) were defined for UV unwrapping. Individual heatmaps were then superimposed to the corresponding 2D UV projection to generate accurate 3D representations (see Fig. 2 for the workflow of the experimental design). All files listed below are provided as supplementary material:

- Please contact the corresponding authors for a “Stand-alone “FlashMap” version 1.0 (see Supplementary software “FlashMap 1.0”) for analysis of complete or subsampled series of brain sections for the generation of complete 2D heatmaps, as well as the averaging of groups of animals.
- Blender (www.blender.org) file template for the generation of 3D heatmaps (see Supplementary Blender file “Template 3D Heatmap”). The template contains a 3D reconstruction of a P10 mouse brain with simplified and unwrapped LV mesh. This template allows straight forward superimposition of 2D expression heatmaps on the 3D model. Further, the file contains a camera fixed to a circular path around the brain for snapshots or 360° movies.
- Example of a 360° movie of a 3D SVZ thickness heatmap (.mp4 file; see Supplementary Movie “3D SVZ Thickness”).

### Software generation – analysis

“FlashMap” was coded using the commercial coding software MATLAB (R2017a; The MathWorks) and a stand-alone version was generated to facilitate distribution and use by other researchers. The stand-alone version directly installs the MATLAB Runtime (v9.2; The MathWorks), which is necessary for proper function of “FlashMap”. “FlashMap” has been tested on Windows 7 and Windows 10. Windows 7 turned out to allow a more stable workflow and is therefore recommended. Description of the workflow for a complete “FlashMap” analysis is described in the current manuscript (see Fig. 3 for an overview of the workflow of the software).

## Electronic supplementary material

Supplementary Figures

Animation
