# Supplementary material for: “FlashMap” - A Semi-Automatic Tool for Rapid and Accurate Spatial Analysis of Marker Expression in the Subventricular Zone
Source: Sci Rep. 2018 Oct 31;8:16086. doi: 10.1038/s41598-018-33939-1 (PMC6208407; doi:10.1038/s41598-018-33939-1)
Supplement: Supplementary file 1 — Supplementary Figures [file 41598_2018_33939_MOESM1_ESM.docx]

**“FlashMap” - A Semi-Automatic Tool for Rapid and Accurate Spatial Analysis of Marker Expression in the Subventricular Zone**

**Stefan Zweifel^1*^, Julie Buquet^1,2,3^, Lorenzo Caruso^1,4^ David Rousseau^2,5^, Olivier Raineteau^1*^**

^1^Univ Lyon, Université Claude Bernard Lyon 1, Inserm, Stem Cell and Brain Research Institute U1208, 69500 Bron, France

^2^CREATIS CNRS UMR5220 & INSERM U1044, Université de Lyon, Université Claude Bernard-Lyon 1, INSA-Lyon, Villeurbanne, France

^3^Institut d’Optique, Université Paris-Saclay, 91127 Palaiseau cedex, France

^4^Télécom Physique Strasbourg, University of Strasbourg, Strasbourg, France

^5^LARIS, IRHS UMR INRA, Université d'Angers, 62 avenue Notre Dame du Lac, 49000, Angers, France

^*^Corresponding authors: [stefan.zweifel86@gmail.com](mailto:stefan.zweifel86@gmail.com); [olivier.raineteau@inserm.fr](mailto:olivier.raineteau@inserm.fr)

**Supplementary Figures**


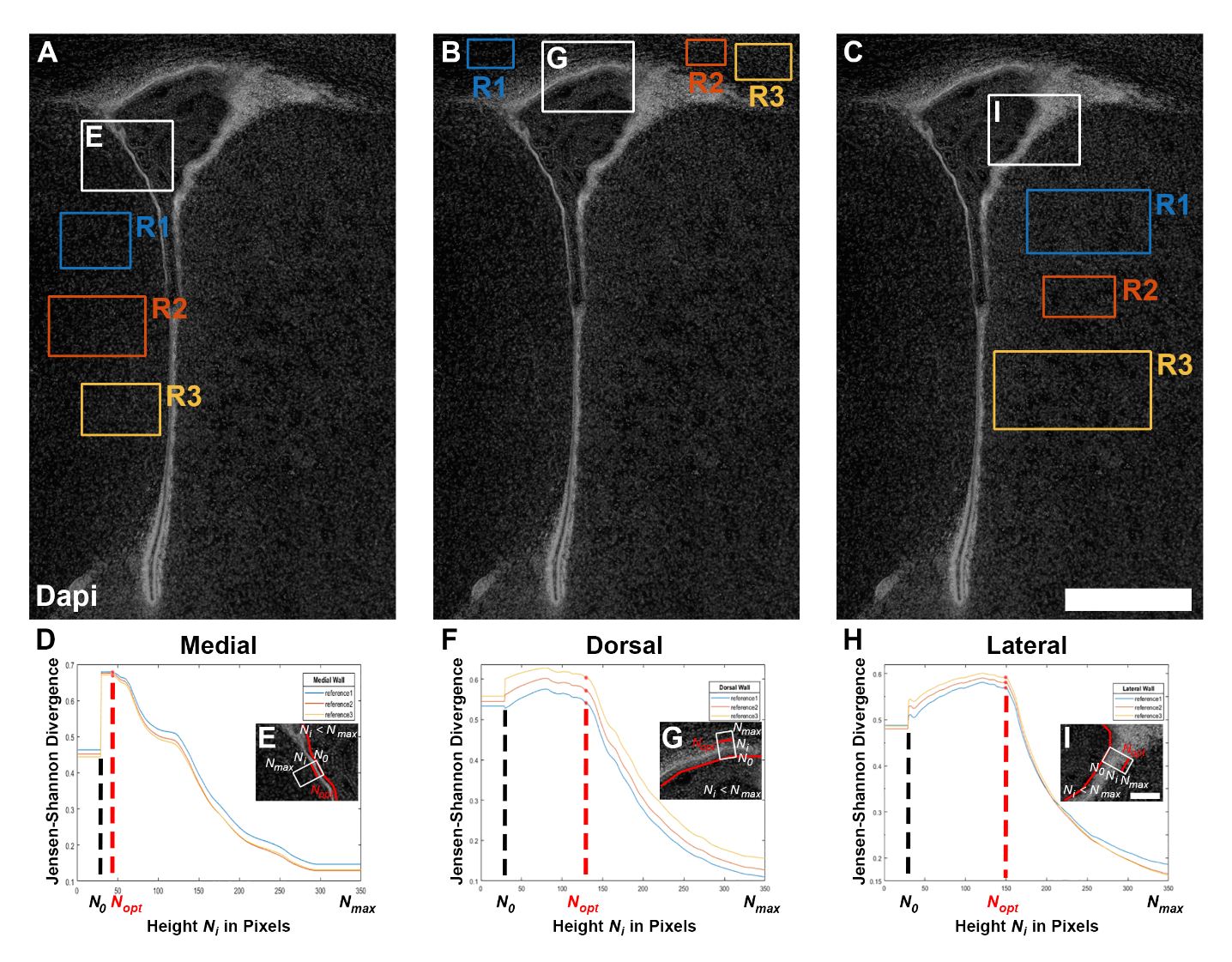


**Figure S1.** Reproducible detection of SVZ thickness with various “reference regions”. **(A-C)** Representative micrographs of Dapi counterstaining for the three ROI: medial (A), dorsal (B), lateral walls (C). Coloured boxes (R1, blue; R2, orange; R3, yellow) represent three different “reference regions” used for automatic detection of probes height (i.e. corresponding to SVZ thickness). **(D-H)** Graphs showing that the precision of the Jensen-Shannon divergence method to define the correct probe height is not affected by selecting different “reference region” size and location. **(E-I)** Representative micrographs illustrating the precision of *N_opt_* calculation, reflected by the optimal positioning of the probe heights. Scale bars: (C) = 500 µm; (I) = 100 µm. Abbreviations: ROI, region of interest; R1-3, “reference region” 1-3.

**
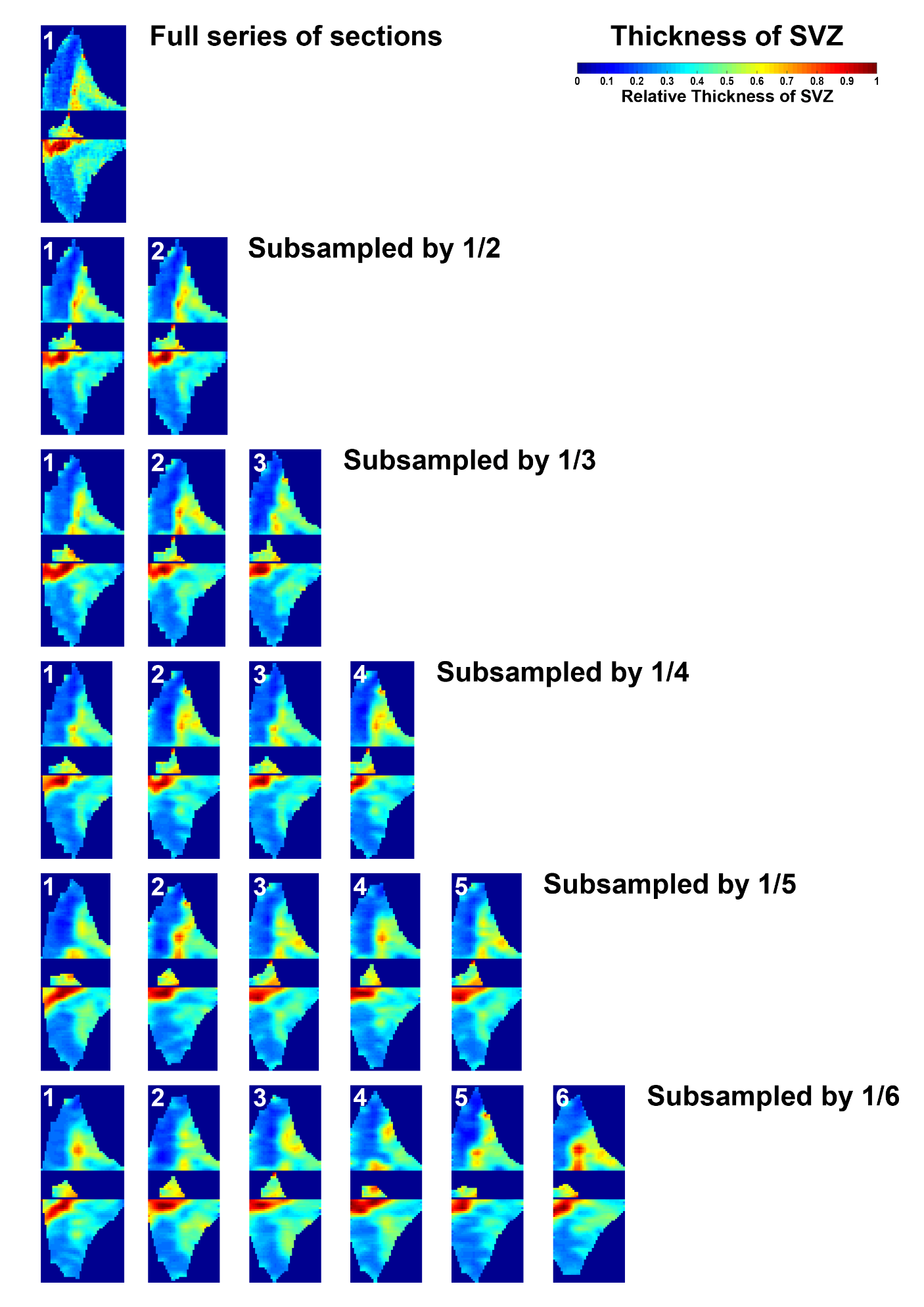

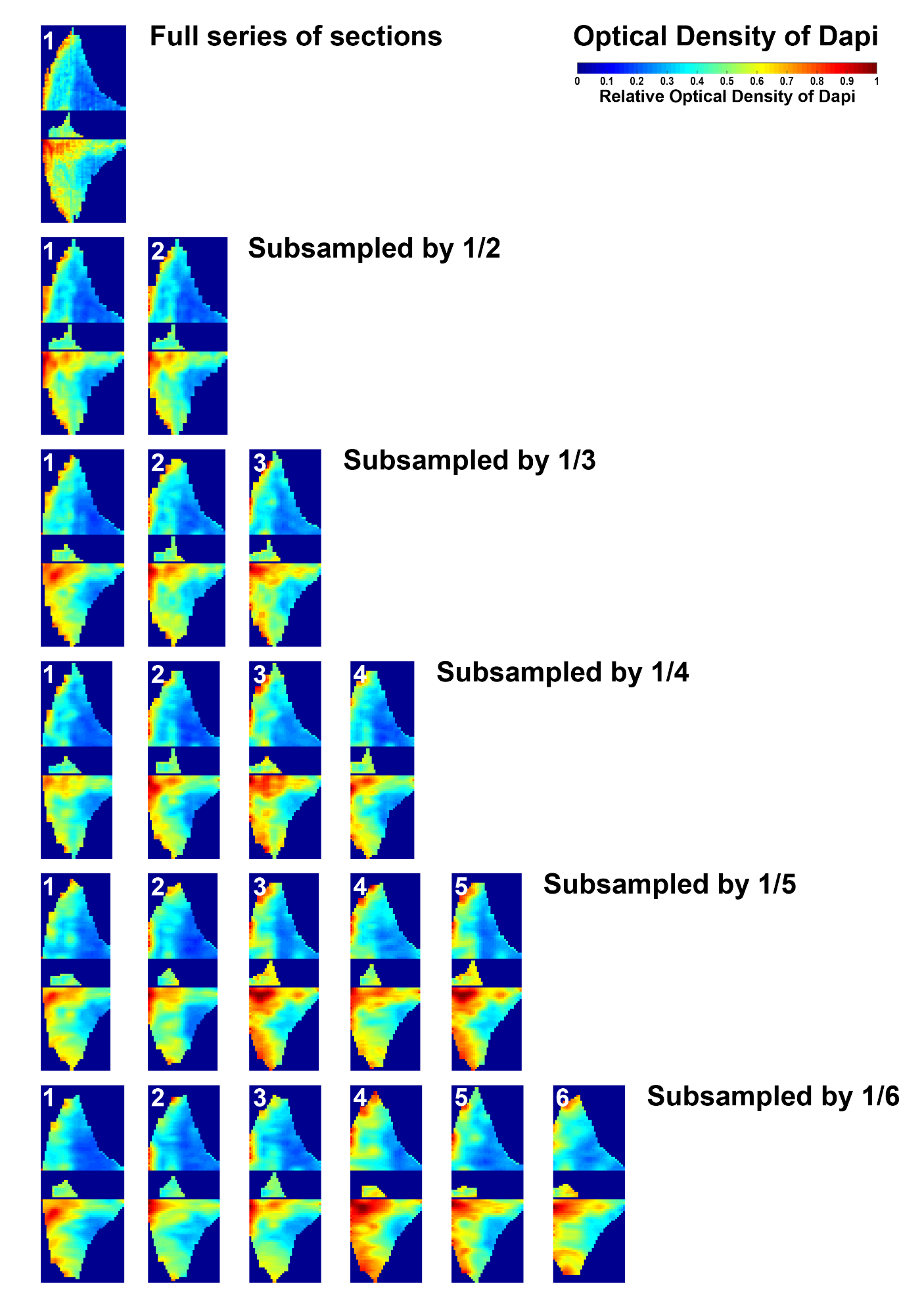

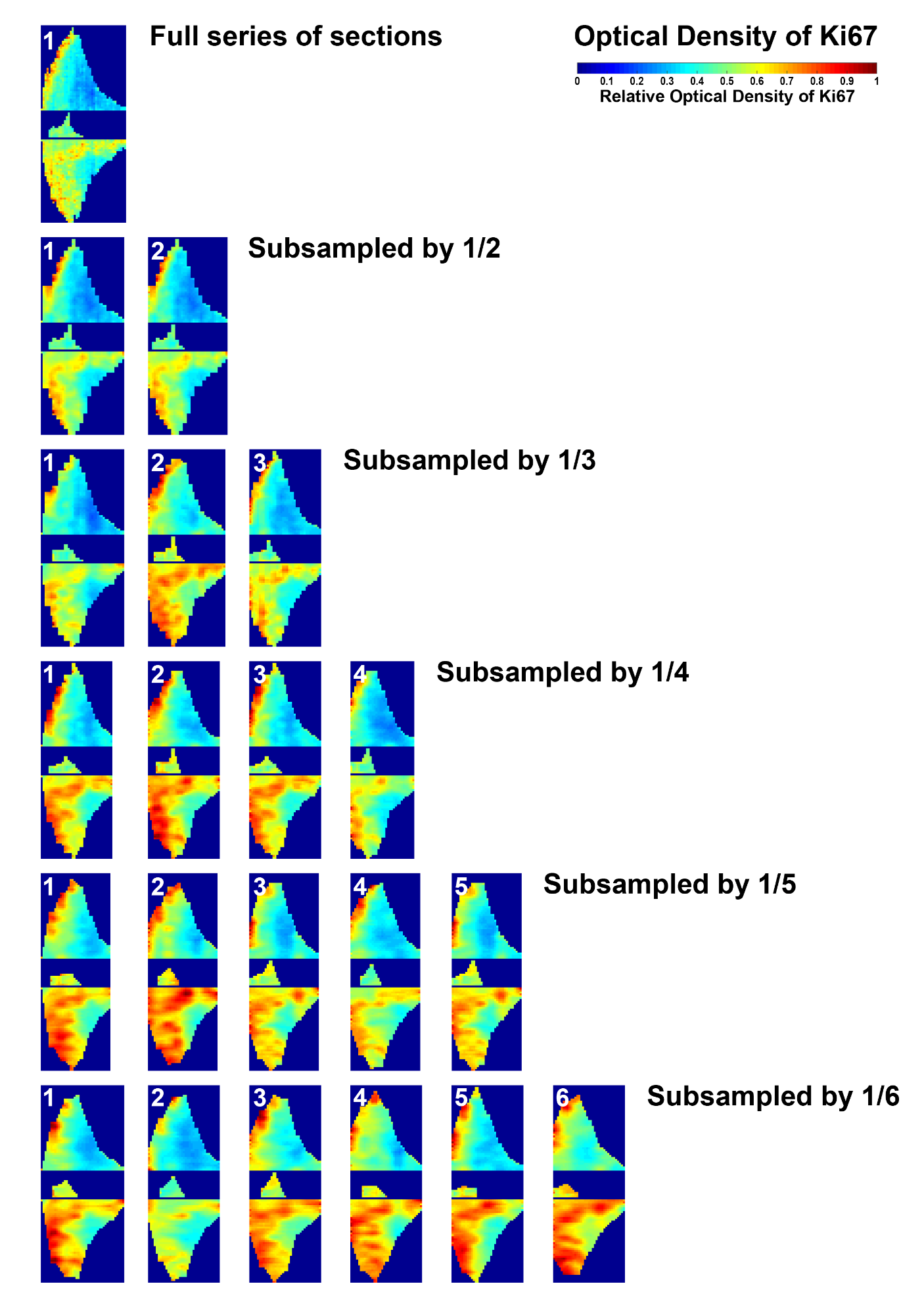
**

**Figure S2 to S4.** Validation of the subsampling approach up to series of 1/6. Heatmaps obtained with subsamplings ranging from 1/2 to 1/6 for analysis of SVZ thickness (Fig. S2), cellular density (Fig. S3) and Ki67^+^ cells density (Fig. S4). Heatmaps of the first line represent full analyses, 2^nd^ to 6^th^ line show the outcome when a subsampling of 1/2 to 1/6 is applied. Row numbers indicate with which section number the analysis was started.

**
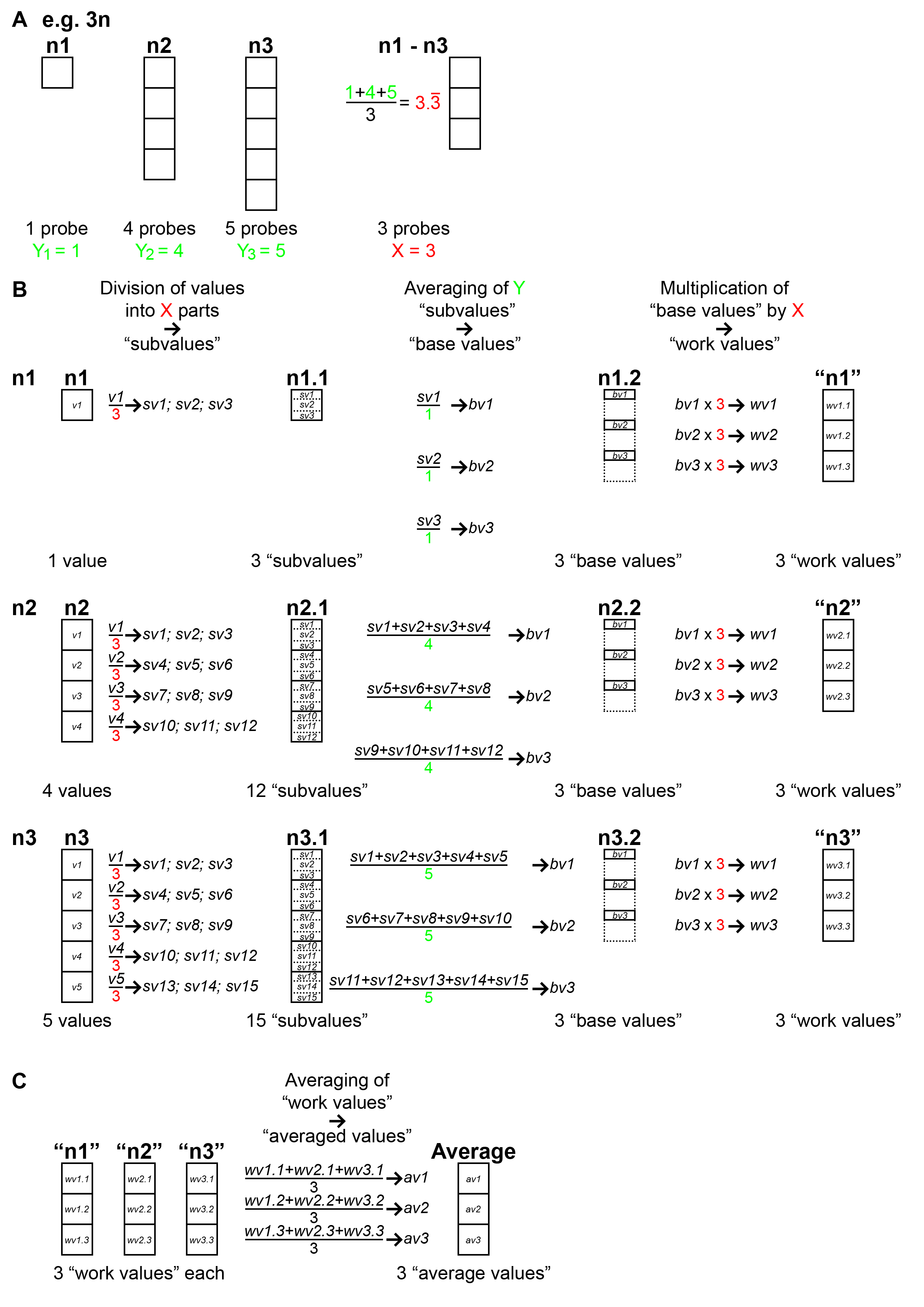
**

**Figure S5.** Detailed illustration of independent analyses averaging, as performed in “FlashMap”. **(A)** In a first step the amount of probes Y_n_ (here Y_1_ = 1; Y_2_ = 4; Y_3_ = 5) are averaged to obtain the final amount of probes X (here 3). **(B)** The values of every ROI and section of each individual (1^st^ panel) are divided by X to obtain Y_n_ * X “subvalues” (2^nd^ panel). The amount of Y_n_ “subvalues” are then averaged, resulting in X “base values” (3^rd^ panel), which are multiplied by X in order to generate X adequate “work values” (4^th^ panel). **(C)** Finally, “FlashMap” averages the corresponding “work values” into X “average values” and generates an averaged map of ODs distribution. Abbreviations: v, value; sv, subvalues; bv, base values; wv, work values; av, average values; OD, optical density.
